# Tocilizumab is associated with reduced delirium and coma in critically ill patients with COVID-19

**DOI:** 10.1038/s41598-024-62505-1

**Published:** 2024-05-23

**Authors:** Tuqa Alkhateeb, Joanna L. Stollings, Ine Sohn, Dandan Liu, L. Montana Fleenor, E. Wesley Ely, Shouri Lahiri

**Affiliations:** 1 The Critical Illness, Brain Dysfunction, and Survivorship (CIBS) Center, Department of Medicine, Division of Allergy, Pulmonary, and Critical Care Medicine, Vanderbilt University Medical Center, Nashville, TN USA; 2 Department of Pharmaceutical Services, Vanderbilt University Medical Center, Nashville, TN USA; 3 Department of Biostatistics, Vanderbilt University Medical Center, Nashville, TN USA; 4 Department of Medicine, Division of Allergy, Pulmonary, and Critical Care Medicine, Vanderbilt University Medical Center, Nashville, TN USA; 5 Geriatric Research, Education and Clinical Center (GRECC) Service, Department of Veterans Affairs Medical Center Tennessee Valley Healthcare System, Nashville, TN USA; 6 Departments of Neurology, Neurosurgery, and Biomedical Sciences, Cedars-Sinai Medical Center, 8700 Beverly Blvd., Los Angeles, CA 90048 USA

**Keywords:** Tocilizumab, Delirium, COVID-19, Sars-CoV-2, ICU, Interleukin-6, Neuroscience, Cognitive neuroscience, Diseases of the nervous system, Neuroimmunology

## Abstract

Recent preclinical studies demonstrate a direct pathological role for the interleukin-6 (IL-6) pathway in mediating structural and functional delirium-like phenotypes in animal models of acute lung injury. Tocilizumab, an IL-6 pathway inhibitor, has shown reduced duration of ventilator dependency and mortality in critically ill patients with COVID-19. In this study, we test the hypothesis that tocilizumab is associated with reduced delirium/coma prevalence in critically ill patients with COVID-19. 253 patients were included in the study cohort, 69 in the tocilizumab group and 184 in the historical control group who did not receive tocilizumab. Delirium was assessed using the Confusion Assessment Method for the Intensive Care Unit (CAM-ICU) with a positive score indicating delirium. Coma was defined as a Richmond Agitation-Sedation Scale score of − 4 or − 5. Tocilizumab was associated with significantly greater number of days alive without delirium/coma (tocilizumab [7 days (IQR: 3–9 days)] vs control [3 days (IQR: 1–8 days)]; p < 0.001). These results remained significant after adjusting for age, sex, sepsis, Charlson Comorbidity Index, Sequential Organ Failure Assessment score, and median daily dose of analgesics/sedatives ($\hat{\upbeta }$ = 0.671, p = 0.010). There were no significant differences in mortality ($\hat{\upbeta }$ = − 0.204, p = 0.561), ventilator duration ($\hat{\upbeta }$ = 0.016, p = 0.956), and ICU or hospital length of stay ($\hat{\upbeta }$ = − 0.134, p = 0.603; $\hat{\upbeta }$ = 0.003, p = 0.991, respectively). Tocilizumab use was associated with significantly increased number of days without delirium/coma. Confirmation of these findings in randomized prospective studies may inform a novel paradigm of pharmacological amelioration of delirium/coma during critical illness.

## Introduction

The severe acute respiratory syndrome coronavirus pandemic has accounted for the admission of tens of millions of patients to intensive care units (ICUs) around the world^1–10^. This severe phenotype of the coronavirus-19 (COVID-19) disease is characterized by acute lung injury and pronounced systemic inflammation, often precipitating the need for mechanical ventilation^11^. In particular, elevated blood concentration of the inflammatory cytokine interleukin-6 (IL-6) is a well-known marker of illness severity and mortality in critically ill patients with COVID-19^12–14^.

Even early in the pandemic, there was widespread recognition of the high rates of delirium experienced by COVID-19 patients admitted to the ICU, occurring in over 80%^15–18^. Various hypotheses were proposed to explain this increased risk including induction of neuroinflammatory mediators, direct viral invasion of the brain, heightened social isolation due to restrictions in family visitations, and the increased need for mechanical ventilation and sedatives; however, no pharmacological treatments have been shown to ameliorate delirium in this patient population^15^.

We recently demonstrated in pre-clinical mouse models of acute lung injury and mechanical ventilation a novel pathological link between elevated peripheral IL-6 levels and delirium-like phenotypes, encompassing both structural and functional brain modifications^19–21^. Our studies suggest that the increased susceptibility of COVID-19 patients to delirium is attributable to the known effect of the virus inducing high levels of circulating IL-6—and that blocking IL-6 activity in the blood may ameliorate delirium.

Tocilizumab, a United States Food and Drug Administration-endorsed IL-6 receptor (IL-6R) antagonist, has been utilized in clinical trials involving early-stage critically ill COVID-19 patients exhibiting signs of systemic inflammation. Trial outcomes have suggested that this IL-6R inhibitor may reduce duration of ventilator dependency and offer survival benefits^22–28^. Consequently, current COVID-19 treatment guidelines advocate the combination of systemic corticosteroids with other immunomodulating agents, such as tocilizumab. However, any potential beneficial effect of tocilizumab on delirium remains unknown.

Accordingly, in this study, we investigate the primary hypothesis that tocilizumab administration to critically ill patients with COVID-19 is associated with significantly increased days of life devoid of delirium or coma.

## Methods

### Study design, participants, and data sources

This was a single-center retrospective cohort study conducted in a tertiary care, academic medical center in the United States. This retrospective study was approved by Vanderbilt University Institutional Review Board (#21240) and were performed in accordance with relevant guidelines and regulations. Patients were included if they were adults 18 years or older and treated in an ICU, with a confirmed diagnosis of COVID-19 infection. We focused only on patients in the ICU, rather than other clinical settings, e.g., ward or ambulatory setting, as patients in the ICU are at higher risk for developing delirium/coma due to a multitude of factors including increased exposure to analgesics/sedatives, increased need for mechanical ventilation, and the ICU environment itself^15^. The tocilizumab group included patients that received concomitant therapy of corticosteroids and tocilizumab from December 1, 2020 to December 31, 2021. The control group (corticosteroids without tocilizumab) included patients that received corticosteroids from July 1, 2020 to November 30, 2020. During this timeframe, tocilizumab was not suggested by the National Institute of Health guidelines as an additional treatment to corticosteroids. Patients were excluded if they were pregnant or prisoners.

### Variables and outcomes

Data collected included age, sex, weight, sepsis diagnosis at admission, Charlson Comorbidity Index at time of encounter, Sequential Organ Failure Assessment (SOFA) score within 48 h of admission, C-reactive protein (CRP) levels within 24 h of admission, ICU days with delirium or coma, ventilator days, length of ICU stay, length of hospital stay, and in-hospital mortality. Due to some patients having missing laboratory values at the day of admission, we used the first available measurement up to a maximum of two days after admission. Also, since IL-6 levels were not routinely measured in clinical care settings before tocilizumab administration in COVID-19 ICU patients, CRP levels were included as CRP has been suggested to act as a surrogate marker for IL-6^29–31^. The SOFA and Charlson Comorbidity Index were calculated from an algorithm with individual components assessed at ICU encounter. Other covariates that were collected included median daily dose of analgesics and sedatives (propofol, opioids, dexmedetomidine, benzodiazepine) during the first 21 days in the ICU. The dose of opioids was converted to fentanyl equivalents. The pharmacy department information technology personnel collected data on ventilator days, length of ICU stay, and length of hospital stay. All other data was manually collected from the electronic medical record.

The primary outcome was the number of calendar days alive without delirium or coma during the first 21 days in the ICU, consistent with prior studies in patients with COVID-19^18,32^. The combined clinical outcome of delirium or coma has been used extensively in paralyzed/sedated patient populations, including critically ill patients with COVID-19^18,33,34^. Delirium and coma were measured until hospital discharge or for up to 21 days in the ICU. Thus, delirium-free and coma-free days were calculated in the 21-day period after ICU admission and were defined as the number of ICU days during which the patient was without delirium and not in coma. Delirium was assessed using the Confusion Assessment Method for the ICU (CAM-ICU) with a positive CAM-ICU score indicating delirium^35^. Coma was defined as a Richmond Agitation-Sedation Scale (RASS) score of − 4 or − 5.

Secondary outcomes included mortality at 90 days, ventilator free days at 21 days, ICU and hospital length of stay, as established outcome measures in critical care studies^36–41^. Ventilator-free days were defined as the number of days alive and breathing without a mechanical ventilator.

### Statistical analysis

Descriptive analyses were done for comparison of baseline characteristics. Wilcoxon test was used for continuous variables and Pearson test for categorical variables. Categorical data are reported as numbers and percentages. Continuous data are reported as medians and interquartile range (IQR).

In the primary analysis, the number of delirium-free and coma-free days was compared between patients who received tocilizumab treatment and patients who received corticosteroids using proportional-odds model, with covariates of age, sex, Charlson Comorbidity Index, SOFA score, sepsis diagnosis at admission, and median daily dose of analgesics and sedatives. No interaction terms were included in the model. The threshold for statistical significance was set at p < 0.05. Proportional-odds assumption was evaluated by plotting logit of cumulative function of response variable versus logarithm of response variable for tocilizumab and control groups.

In the secondary analyses, proportional-odds models were used to assess the difference in ICU length of stay in days, hospital length of stay in days, and ventilator-free days, respectively, between the tocilizumab group and control group. Binary logistic regression was used to assess the 90-days mortality rate between the tocilizumab group and control group. For all four analyses, the same independent covariates used in primary analysis was used. All analyses were performed using R Statistical Software (R version 4.2.2 (2022-10-31 ucrt); R Core Team 2022). For statistical analysis, Hmisc package (v5.1-0; Harrell 2023) and rms package (v6.7-0; Harrell 2023) were used. Forest plot was created using forestplot package (v3.1.3; Gordon 2023).

### Ethics approval and consent to participate

This need for informed consent is waived by the Vanderbilt Institutional Review Board (#212409).

## Results

**Figure 1:**
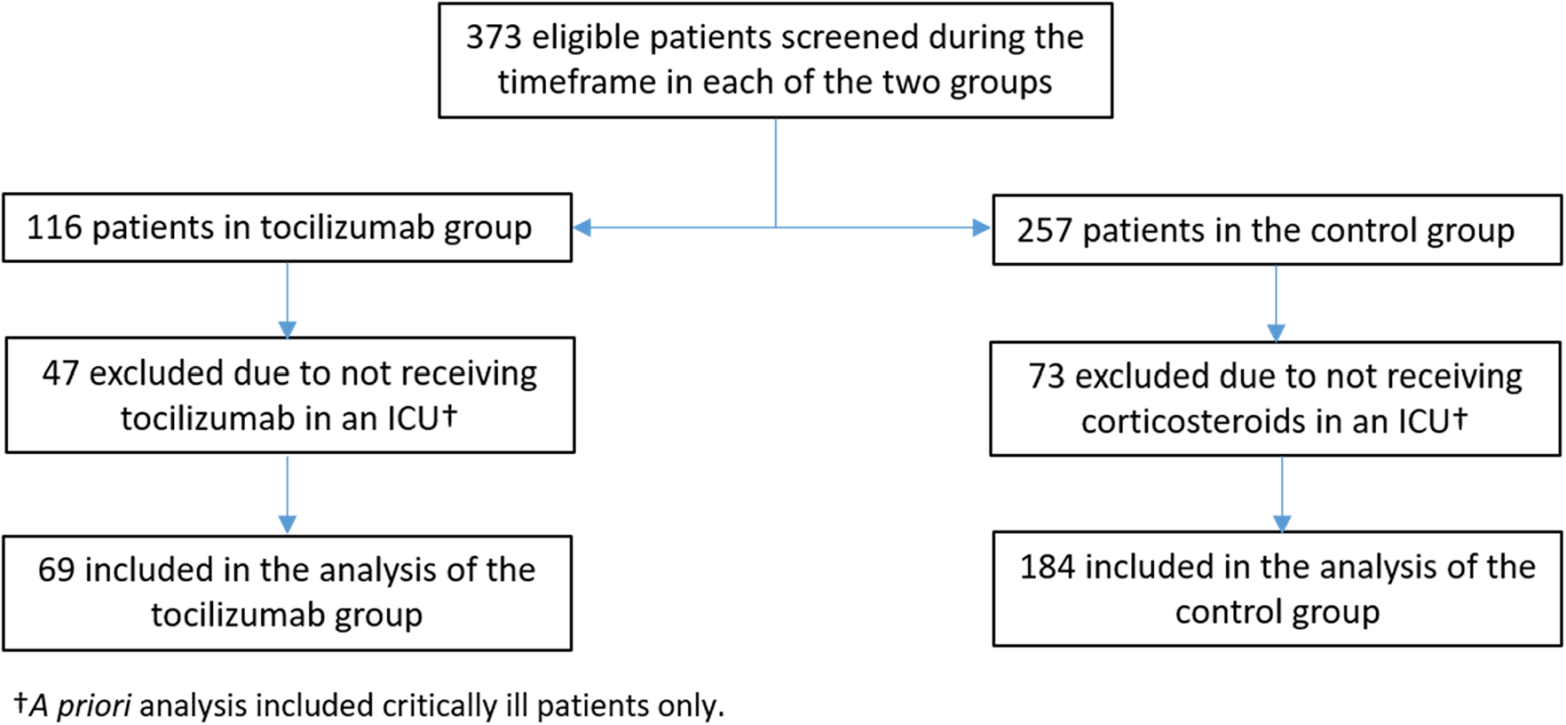
Flow diagram demonstrating screening process and selection of patients for inclusion in study.

A total of 373 patients were consecutively screened for eligibility. 116 patients were admitted between December 1, 2020 and December 31, 2021 after recommendation for tocilizumab use was published, i.e., “tocilizumab group”, and 257 patients were admitted between July 1, 2020 and November 30, 2020, i.e. “control group”. Of these, 47 patients were excluded from the tocilizumab group as they did not receive tocilizumab in an ICU and 73 patients were excluded from the control group as they did not receive corticosteroids in an ICU. Thus, 253 patients were included in the study cohort, with 69 patients in the tocilizumab group and 184 patients in the control group (Fig. 1).

In the overall cohort, the median age was 61 years (IQR: 52–70 years), and the majority were male (62%, n = 158). Baseline comparisons between the groups were similar overall (Table 1). However, age and the Charlson Comorbidity Index at time of encounter were lower in the tocilizumab group. There were no significant differences between groups in the usage of analgesics or sedatives (Table 2).

**Table 1:** Baseline characteristics.

	Tocilizumab group (n = 69)	Control group (n = 184)	p value
Age, yr, median (IQR)	55 (44, 65)	63 (54, 71)	**< 0.001**
Male, n (%)	37 (54)	121 (66)	0.076
CCI at encounter, median (IQR)	2 (1, 3)	3 (2, 5)	**< 0.001**
SOFA score on admission, median (IQR)	5 (3, 6)	5 (4, 8)	0.204
Presence of sepsis on admission, n (%)	15 (22)	32 (17)	0.428
CRP on admission, mg/l, median (IQR)*	137 (91, 203)	132 (68, 210)	0.318

*n = 214 due to missing CRP values in both groups. Significant values are in bold.

*CCI* Charlson Comorbidity Index, *CRP* C-reactive protein, *IQR* interquartile range, *l* liters, *mg* milligrams, *SOFA* Sequential Organ Failure Assessment, *yr* years.

**Table 2:** Analgesia and sedative usage.

	Tocilizumab group (n = 69)	Control group (n = 184)	p value
Dexmedetomidine exposure			
Ever used, n (%)	33 (48)	87 (47)	0.939
Median days among users, median (IQR)	3 (2, 5)	3 (2, 6)	0.618
Median daily dose on days administered, μg/kg/h, median (IQR)	0.5 (0.2, 0.8)	0.4 (0.2, 0.8)	0.960
Midazolam exposure			
Ever used, n (%)	23 (33)	48 (26)	0.253
Median days among users, median (IQR)	2 (1, 6)	4 (1, 10)	0.284
Median daily dose on days administered, mg/day, median (IQR)	89.7 (22.0, 151.4)	99.7 (39.2, 190.8)	0.394
Opioids exposure			
Ever used, n (%)	41 (59)	110 (60)	0.958
Median days among users, median (IQR)	7 (5, 11)	7 (2, 11)	0.339
Median daily dose on days administered, μg/h, median (IQR)*	237 (160.4, 314)	210 (90.2, 320)	0.309
Propofol exposure			
Ever used, n (%)	41 (59)	105 (57)	0.736
Median days among users, median (IQR)	5 (3, 8)	5 (3, 8)	0.752
Median daily dose on days administered, μg/kg/min, median (IQR)	28 (20, 35)	21 (14, 33)	0.085

*Values shown are in intravenous fentanyl equivalents.

*h* hour, *IQR* interquartile range, *kg* kilograms, *μg* micrograms, *min* minute.

Tocilizumab one-time dosage administration was either 800 mg or 8 mg/kg with the median dose administered as 800 mg (IQR: 720–800 mg). Tocilizumab was administered within 1 day of hospital admission in 57/69 (83%) patients and within 1 day of ICU admission in 61/69 (88%) patients. In the tocilizumab group, corticosteroid administration was initiated within 1 day of hospital admission in 61/69 (88%) of patients and within 1 day of ICU admission in 62/69 (90%) of patients. Corticosteroids were used for a median of 9 days (IQR: 7–11 days) in this group and the cumulative median total corticosteroid dose (dexamethasone equivalents) used was 54 mg (IQR: 42–84 mg).

In the control group, 168/184 (91%) of patients received dexamethasone 6 mg including 16/168 (10%) of those patients receiving additional corticosteroids (hydrocortisone, methylprednisolone, and/or prednisone) during their hospital stay. The other patients in the control group 16/184 (9%) received non-dexamethasone corticosteroids such as hydrocortisone, methylprednisolone, and/or prednisone during their hospital stay. Corticosteroids were administered within 1 day of hospital admission in 147/184 (80%) of patients and within 1 day of ICU admission in 155/184 (84%) of patients. Corticosteroids were used for a median of 8 days (IQR: 5–9 days) in this group and the cumulative median total corticosteroid dose (dexamethasone equivalents) used was 48 mg (IQR: 30–54 mg).

The median days alive without delirium or coma were 7 days (IQR: 3–9 days) in the tocilizumab group and 3 days (IQR: 1–8 days) in the control group (p < 0.001). SOFA scores were the only covariate determined to affect this primary outcome, in that patients with lower SOFA scores at admission had more days free without delirium or coma. Delirium or coma occurred in 44/69 (64%) of patients in the tocilizumab group and 134/184 (73%) of patients in the control group (p = 0.16).

There was no difference between median ventilator-free days between the tocilizumab and control groups (2.77 [0–10.7] vs 0.583 days [IQR: 0–8.3 days]; p = 0.539). From the 40 (58%) of 69 tocilizumab patients that were on mechanical ventilation, 27 (68%) of those 40 died. Similarly, from the 110 (60%) of 184 control patients that were on mechanical ventilation, 72 (65%) of those 110 patients died. There was a non-statistically significant trend towards improved 90-day mortality in tocilizumab-treated patients and no differences in ventilator free days, ICU length of stay, and hospital length of stay (Table 3).

**Table 3:** Primary and secondary efficacy end points.

	Tocilizumab group (n = 69)	Control group (n = 184)	p value
Primary end point			
Delirium/comatose occurrence, n (%)	44 (64)	134 (73)	0.16
Days alive without delirium/coma at 21 days, median (IQR)	7 (3, 9)	3 (1, 8)	**< 0.001**
Secondary end points			
Ventilator-free days at 21 days, median (IQR)	2.7 (0, 10.7)	0.58 (0, 8.25)	0.539
Death at 90 days, n (%)	32 (46)	106 (58)	0.11
Days of ICU stay, median (IQR)	9.8 (6.2, 16.5)	8.9 (4.1, 17.6)	0.529
Days of hospital stay, median (IQR)	13.8 (9.2, 25.6)	12.7 (8.1, 23.0)	0.338

Significant values are in bold.

*IQR* interquartile range.

**Figure 2:**
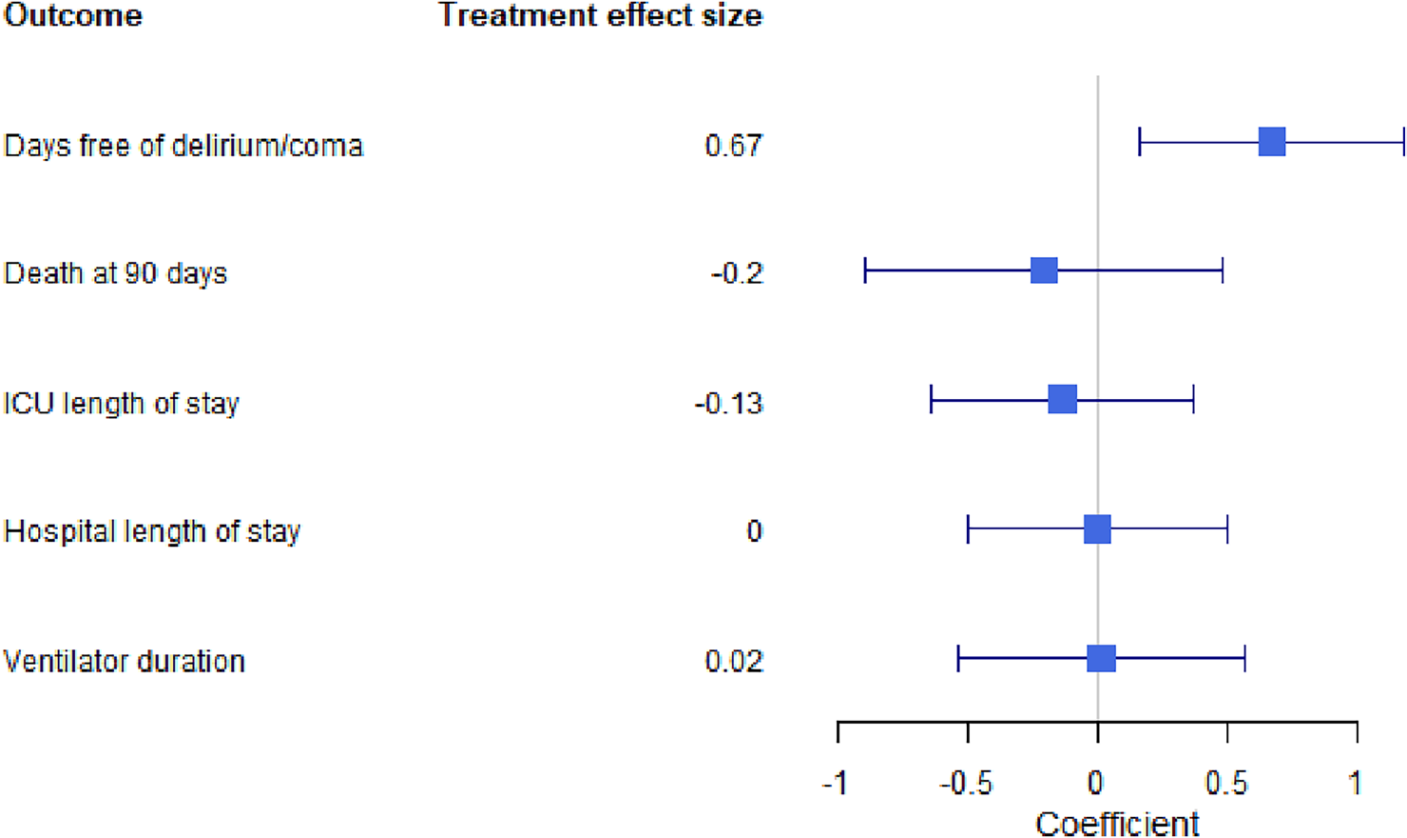
Forest plot showing effect size of tocilizumab on days free of delirium/coma, death at 90 days, ICU length of stay, hospital length of stay, and ventilator duration. Positive coefficient values indicate favorable effect of tocilizumab on delirium/coma while negative coefficient values indicate favorable effect of tocilizumab on death at 90 days, ICU length of stay, hospital length of stay, ventilator duration.

In proportional odds logistic regression analysis, after accounting for age, sex, SOFA, Charlson Comorbidity Index, sepsis, dexmedetomidine, midazolam, opioids, or propofol use, patients treated with tocilizumab and corticosteroids remained significantly more likely to experience increased delirium- or coma-free days compared to patients treated with corticosteroids alone ($\hat{\upbeta }$ = 0.671, p = 0.010) (Fig. 2 forest plots). In contrast, there were no significant differences in mortality ($\hat{\upbeta }$ = − 0.204, p = 0.561), ventilator duration ($\hat{\upbeta }$ = 0.016, p = 0.956), ICU length of stay ($\hat{\upbeta }$ = − 0.134, p = 0.603), or hospital length of stay ($\hat{\upbeta }$ = 0.003, p = 0.991) (Fig. 2 forest plots).

## Discussion

To our knowledge, this is the first study to demonstrate an association between use of an IL-6 pathway inhibitor, tocilizumab, and reduced delirium or coma in critical illness. Our findings indicate that patients with severe COVID-19 who received tocilizumab alongside corticosteroids experienced a significantly higher number of delirium- or coma-free days in comparison to those who were only treated with corticosteroids.

Several theories have postulated a connection between delirium, systemic inflammation, and brain inflammation. Our recent preclinical studies using murine models of delirium in mechanical ventilation and acute lung injury demonstrate a direct pathophysiological role for peripheral IL-6 in inducing delirium-like structural and functional phenotypes via the IL-6-*trans*-signaling pathway^19–21^. In this pathway that has been implicated in diverse neurodegenerative conditions, IL-6 binds to soluble IL-6R in the periphery and directly induces neuronal injury via glycoprotein 130 without needing to bind to any classic membrane receptors, which neurons lack. The findings from this clinical study are consistent with these prior preclinical findings implicating IL-6-*trans*-signaling as a principal pathway in delirium pathogenesis. However, it remains possible that delirium/coma reduction in this clinical study was achieved via tocilizumab’s anti-inflammatory effects on other organs such as the liver, resulting in improved metabolism of sedatives like midazolam. Future studies are indicated to evaluate the contributions of these distinct pathways to delirium/coma duration.

Mechanical ventilation and acute lung injury of varied etiologies are well-established risk factors for delirium^29,42,43^, with this and prior clinical studies demonstrating > 50% prevalence of delirium/coma in this population^18^. Indeed, several clinical studies have demonstrated strong associations between peripheral IL-6 and delirium/coma in mechanically ventilated patients with acute lung injury^19,44,45^.

Although peripheral IL-6 levels were not available to facilitate a comparison of inflammation at admission between the groups, the overall CRP levels, which act as a surrogate marker for IL-6, demonstrated no significant disparity within the first 24 h of admission between the cohorts, indicating similar severities of systemic inflammation between the two groups. Intriguingly, in the tocilizumab group, median CRP levels did not appear to influence survival outcomes, and CRP levels were slightly more elevated in patients who survived (149.5 mg/l [IQR: 112–228] vs 132 mg/l [IQR: 82–200]; p = 0.25) congruent with earlier studies suggesting a protective survival effect for tocilizumab in patients presenting with higher CRP levels upon admission^9,27,46^.

Our study findings extend the previously reported survival benefits of tocilizumab and corticosteroids to now include the potential for significantly reduced delirium or coma episodes in patients with severe COVID-19^27,28^. Though incongruent with prior clinical trials, the lack of improvements in survival or ventilator duration among tocilizumab-treated patients in our study supports an independent beneficial effect of peripheral IL-6 antagonism on delirium/coma outcomes. However, as prior studies that showed improvements in survival and ventilator duration had larger sample sizes it remains possible that our study was not adequately powered to detect improvements in survival or ventilator duration, despite being sufficiently powered to detect a difference in delirium/coma duration.

A recent study with a focus on long-term neuropsychological assessments and subjective physical and mental wellbeing in tocilizumab-treated patients with COVID-19 found a non-statistically significant trend towards improved delirium or coma-free days among tocilizumab and corticosteroid-treated compared to corticosteroid-only treated patients (p = 0.098)^47^. This study had a higher proportion of mechanically ventilated patients and may have been underpowered to detect significant differences in delirium/coma due a smaller cohort (n = 26–42/group) than our study and did not adjust for covariates known to affect delirium/coma risk including baseline differences in CRP, benzodiazepine, opiate, or dexmedetomidine use^47^. Furthermore, as days of mechanical ventilation was significantly lower in the tocilizumab and corticosteroid compared to the corticosteroid only group it is possible that reduced exposure to mechanical ventilation and associated components, such as sedatives, accounted for the differences in delirium/coma rather than IL-6-mediated brain dysfunction—unlike in our study that showed no significant differences in duration of mechanical ventilation. Nevertheless, future studies are needed to evaluate whether any improvements in delirium or coma conferred by tocilizumab translate to longer-term cognitive benefits.

This study has several notable strengths. First, it is supported by extensive preclinical studies that provide a strong biological rationale for the observed findings. Second, our analysis takes into account a multitude of covariates, recognized as robust predictors of our outcome measures. Finally, although this study focused on patients with COVID-19, as peripheral IL-6 levels are increased in diverse infectious and non-infectious systemic conditions, this study lays the foundation for future phase II studies that modulate the IL-6 pathway to ameliorate delirium in varied clinical scenarios.

Our study is not without limitations—it is inherently retrospective, single-center and reliant on charted documented assessments for delirium/coma diagnosis. We were unable to consider additional concurrent therapies beyond the scope of analgesics, sedatives, steroids, and/or tocilizumab. The measurement of CRP levels may not accurately mirror the pre-tocilizumab inflammatory state of patients as most patients (≥ 80%) in our study that received tocilizumab had CRP levels assessed within a day of hospital admission. Additionally, the influence of patients' vaccination status, the implications of different COVID-19 variants, and evolutions in processes of care over the study period, all potential modifiers of the inflammatory and/or delirium outcome, were not taken into consideration. We further recognize that it is difficult to completely disentangle direct effects of tocilizumab on delirium/coma from an indirect salutary effect given the drug’s known benefit effects on recovery and mortality in COVID-19. Furthermore, although similar baseline SOFA scores, presence of sepsis, C-reactive protein levels indicate similar severity of acute illness, we did not have data on education status, insurance/socioeconomic status, PaO2:FiO_2_ ratio, vasopressor utilization, diagnosis of acute renal failure, or any other non-Sars-CoV-2 etiologies of sepsis—future prospective or propensity-matched design studies are indicated to evaluate any contributions of these various factors to delirium or coma and confirm our findings. Nonetheless, the potential novel mechanism of tocilizumab in reducing delirium or coma is grounded in extensive preclinical work and may inform future pharmacological therapies for delirium, which are currently severely lacking^33,48^.

## Conclusions

This study’s findings indicate an association between tocilizumab administration and reduced delirium/coma in critically ill patients with severe COVID-19. Confirmation of these findings in randomized prospective studies could lead to a novel paradigm of pharmacological amelioration of delirium/coma during critical illness.

## Data Availability

The datasets used and/or analyzed during the current study are available from the authors on reasonable request.

## Abbreviations

ICUs: Intensive care units
COVID-19: Coronavirus-19
IL-6: Interleukin-6
IL-6R: Interleukin-6 receptor
SOFA: Sequential Organ Failure Assessment
CRP: C-reactive protein
CAM-ICU: Confusion Assessment Method for the ICU
RASS: Richmond Agitation-Sedation Scale
IQR: Interquartile range
